# A comparative study of cardiovascular risk stratification methods in type 1 diabetes mellitus patients

**DOI:** 10.1186/s13098-023-01224-5

**Published:** 2024-01-09

**Authors:** Fabiano Malard de Araujo, Fábio Vasconcellos Comim, Rodrigo N. Lamounier, Natália F. Pena, Virgínia C. Fajardo, Regina Bicalho G. de Faria, Yuri M. Silva, Márcio Weissheimer Lauria

**Affiliations:** 1 Hospital das Clínicas da Universidade Federal de Minas Gerais, Belo Horizonte-MG, Brazil; 2 Universidade Federal de Minas Gerais, Rua São Paulo 1755, apt. 1203, Belo Horizonte-MG, 30170-135 Brazil

**Keywords:** Cardiovascular risk, Type 1 diabetes mellitus, Steno calculator, ST1RE

## Abstract

The Steno Diabetes Center Copenhagen developed the Steno T1 Risk Engine (ST1RE) to predict cardiovascular events, encompassing fatal and nonfatal ischemic heart disease, ischemic stroke, heart failure, and peripheral arterial disease in type 1 diabetes mellitus(T1DM).

The current study investigated the agreement between ST1RE and the Brazilian Society for Endocrinology and Metabology (SBEM) classification. Participants were included in the study if diagnosed with T1DM and had at least one outpatient visit in 2021. Patients with established cardiovascular disease and chronic kidney disease on dialysis were excluded. Clinical parameters were obtained from medical records, such as age, body mass index (BMI), blood pressure, physical activity, current smoking, microvascular target organ damage, levels of low-density lipoprotein cholesterol, creatinine, glycated hemoglobin (HbA1c), and albuminuria.

Overall, 92 patients (38 males and 53 females) with an age median (P25; P75) of 33 years (25.5;42.5), BMI of 24.8 + 4.1 kg/m2, and duration of diabetes (mean ± SD) of 23.4 + 9.5 years were evaluated. There were no differences considering the gender for most analyzed variables, but a higher proportion of women exhibited microvascular complications such as microalbuminuria, macroalbuminuria, and retinopathy. Our results show a weak agreement in the 10-year cardiovascular risk estimation between SBEM and ST1RE classifications. According to SBEM criteria, 72.8% of patients were considered high-risk, while only 15.2% of patients received the same classification using ST1RE. The dissimilarities between these two classifications were also evident when age and gender factors were compared. While 60% of patients under 35 years were classified as high risk according to SBEM criteria, only 1.8% received this stratification risk in the ST1RE classification.

The results indicate a low agreement between the 10-year cardiovascular event risk classification by SBEM and the classification by ST1RE for type 1 diabetes patients without established cardiovascular disease.

## Introduction

Despite access to appropriate treatment, type 1 diabetes mellitus (T1DM) patients exhibit a mortality rate approximately three times higher than the general population, with cardiovascular diseases emerging as the primary cause [1]. Evidence from the literature points that the probability of cardiovascular events in T1DM is significantly elevated as if the patients were 10 to 15 years older, being the difference higher in women than in men [2]. This risk becomes even more relevant as life expectancy of diabetic patients’ post-diagnosis increases [3].Notably, conventional risk assessment tools such as the Atherosclerotic Cardiovascular Disease (ASCVD) equation demonstrate limited applicability for T1DM patients, failing to consider crucial factors like age of diagnosis, disease duration [4], proteinuria, microalbuminuria [5, 6], and glycemic control [7–9]. Similarly, the employment of UKPDS calculator, based on the results of The United Kingdom Prospective Diabetes Study which included these factors miscalculated the cardiovascular risk for T1DM subjects [10, 11]. Some factors such as gender and glycemic control may operate distinctively between T1DM and in type 2 diabetes mellitus (T2DM) [12, 13].

Consequently, the need for tailored risk stratification methods specific to T1DM patients becomes evident. To address this gap, the Steno Diabetes Center Copenhagen developed the Steno T1 Risk Engine (ST1RE) [14–16]. This tool aims to enhance precision in cardiovascular risk estimates for T1DM patients. Utilizing data from a tertiary center in Denmark spanning 2001 to 2013, the algorithm was devised to predict first cardiovascular event, encompassing fatal and non-fatal episodes of ischemic heart disease, ischemic stroke, heart failure, and peripheral arterial disease. While the ST1RE tool has demonstrated promise, a limitation lies in its exclusive reliance on Danish population data, albeit publications of studies in other countries. Further investigations employing the ST1RE tool have revealed correlations with pre-clinical outcomes such as atherosclerosis and arterial stiffness [15, 16]. However, divergent outcomes were observed in an Italian study, where the ST1RE tool significantly overestimated absolute cardiovascular event risk [17]. Additionally, a study comparing the ST1RE tool with the European Society of Cardiology’s 2019 risk classification showed variations in high cardiovascular risk stratification proportions, underscoring the imperative to refine risk assessment tools for optimal preventive interventions [18]. Ongoing efforts seek to enhance ST1RE’s performance by considering supplementary parameters like echocardiographic evaluation and NT-proBNP serum levels [19, 20]. In Brazil, risk stratification methods for T1DM patients align with those for T2DM patients, despite distinctive risk profiles [21]. While the latest guidelines from the Brazilian Society of Diabetes (SBD) incorporate the ST1RE tool for patients with less than two decades of disease duration and without other high-risk indicators, the critiques apply to these methodologies [22].

Therefore, the present study aims to apply the novel ST1RE risk stratification tool and compare it with existing methodologies endorsed by the Brazilian Society of Endocrinology and Metabolism (SBEM) [21]. By assessing the concordance between these approaches and identifying disparities, this research aims to elucidate the strengths and limitations of these methodologies, thereby guiding their clinical application.

## Materials and methods

### Study design

This was a cross-sectional study, from retrospective medical records of patients attending outpatient diabetic clinics at Hospital das Clínicas, Federal University of Minas Gerais (UFMG), Brazil. Participants were included in the study if diagnosed with T1DM and had at least one outpatient visit in 2021. Patients with established cardiovascular disease, chronic kidney disease on dialysis, or incomplete laboratory tests were excluded from this study.

### Data collection and assessment of the cardiovascular risk

Clinical parameters, such as age, weight, height, body mass index (BMI), abdominal circumference, and blood pressure were evaluated in all patients. Regular physical activity was a dichotomous variable, indicating positive response for patients engaged in ≥ 3.5 h of weekly activity. Current smoking was defined as consuming one or more cigarettes per day. Microvascular target organ damage encompassed diabetic retinopathy, nephropathy, and neuropathy. Retinopathy was defined through specific ophthalmological evaluation, while nephropathy was identified by reduced creatinine clearance (< 60 ml/min/1.73 m²) and/or presence of micro or macroalbuminuria. Neuropathy diagnosis relied on clinical assessment, including symptom evaluation and clinical examination, without supplementary exams. Laboratory parameters included the serum levels of low-density lipoprotein (LDL) cholesterol, creatinine, glycated hemoglobin (HbA1c), and urinary levels of albuminuria. The glomerular filtration rate was estimated using the Chronic Kidney Disease Epidemiology Collaboration (CKD-EPI) formula continuous variables. Cardiovascular risk was estimated for all patients using both the ST1RE tool and recommendations from the SBEM guideline. Patients were categorized into low, intermediate, and high/very high-risk groups by both methods.

### Statistical analysis

Data were presented as mean ± standard deviation (SD) or median and percentiles (P) 25–75 following graphical analysis for normality. Categorical data were expressed as absolute frequency (relative frequency; %). Clinical parameters were analyzed for the presence of statistical differences between males and females in Table 1, and between high risk population by SBEM and high risk population by ST1RE in Table  2. Continuous data were analyzed using t-Student and Mann-Whitney U tests based on data normality, while categorical data were assessed using Pearson’s chi-square or Fisher’s exact tests as displayed in Tables 1 and 2. Cohen’s Kappa test was employed to calculate agreement between the two methods [23]. The tools were considered to be in agreement when the same patient received an equal cardiovascular risk classification – low, intermediate or high – by both tools.

**Table 1:** General characteristics of the study population

	Total N = 92	Men (n = 38)	Women (n = 53)	p-value
Age (years)	33.0 (25.5; 42.5)	33.0 (26.0; 40.0)	33.0 (25.0; 43.0)	0.955
Diabetes duration (years)	23.4 (± 9.5)	22.9 (± 9.0)	23.7 (± 10.0)	0.685
Age of T1DM onset (years)	11.0 (5.0; 15.0)	10.0 (4.0; 15.0)	11.0 (5.0; 15.0)	0.955
Early onset T1DM (1–10 years of age)	47.8 (44)	52.6 (20)	45.3(24)	0.479
BMI (kg/m^2^)	24.8 (± 4.1)	24.9 (± 3.2)	24.6 (± 4.7)	0.790
HbA1c (%)	8.3 (7.5; 9.7)	8.2 (7.4; 9.3)	8.8 (7.6; 10.1)	0.101
SBP (mmHg)	122.3 (± 15.6)	121.1 (± 11.7)	123.0 (± 18.0)	0.580
DBP (mmHg)	75.6 (± 10.2)	75.1 (± 10.0)	75.9 (± 10.5)	0.716
LDL-cholesterol (mg/dl)	94.9 (± 27.2)	91.6 (± 25.3)	97.7 (± 28.5)	0.295
eGFR (CKD-EPI) (ml/min/1.73 m^2^)	105.5 (80.5; 117.0)	111.5 (94.0; 117.0)	99.0 (75.0; 116.0)	0.084
Serum creatinin (mg/dL)	0.82 (0.75; 0.95)	0.90 (0.79; 0.97)	0.78 (0.66; 0.94)	**0.007**
Albumin-Creatinin Ratio (mg/g)				
Normal	65.2 (60)	79.0(30)	56.6 (30)	**0.035**
Microalbuminúria	17.4 (16)	5.2 (2)	24.5 (13)	
Macroalbuminúria	17.4 (16)	15.8 (6)	18.9 (10)	
Microvascular complications (%)	62.0 (57)	47.4 (38)	71.7 (38)	**0.019**
Nephropathy (%)	40.2 (37)	29.0 (11)	47.2 (25)	0.080
Retinopathy (%)	54.4 (50)	39.5 (15)	64.2 (34)	**0.020**
Neuropathy (%)	10.9 (10)	5.3 (2)	13.2 (7)	0.295
Treatments				
Statins	39.1 (36)	39.6 (21)	36.8 (14)	0.788
ACE inhibitor or ARB	48.9 (45)	52.8 (28)	42.1 (16)	0.313
AAS	5.4 (5)	7.9 (3)	1.9 (1)	0.304
Physical activity	14.1 (13)	18.4 (7)	11.3 (6)	0.375
Smoking	5.8 (5)	10.8 (4)	2.1 (1)	0.162

*Data are expressed as mean (± standard deviation) or median (P25;P75) or as a percentage (%); SBP systolic blood pressure; DBP diastolic blood pressure; eGFR estimated glomerular filtration rate; BMI body mass index; T1DM type 1 diabetes mellitus; HBA1c glycated hemoglobin*

## Results

The key characteristics of the studied population (n = 92; 39 males and 53 females) are displayed in Table 1. The median (P25; P75) for age was 33 years (25.5; 42.5), and HBA1c was 8.3% (7.5;9.7). The BMI was (mean + SD) of 24.8 + 4.1 kg/m^2^, and the duration of diabetes was (mean + SD) 23.4 + 9.5 years. Systolic blood pressure was (mean + SD) 122.3 + 15.6 mmHg and diastolic of 75.6 + 10.2 mmHg. The LDL cholesterol levels (mean + SD) was 94.3 + 27.2 mg/dL. There were no differences considering the gender for most analyzed variables such as age, duration of diabetes, age at the time of diagnosis, BMI, tobacco use, HBA1c, systolic and diastolic blood pressure, LDL cholesterol, and creatinine clearance. However, a higher proportion of women exhibited microvascular complications such as microalbuminuria, macroalbuminuria, and retinopathy (Table 1). The prevalence of neuropathy was similar between the genders.

**Fig. 1:**
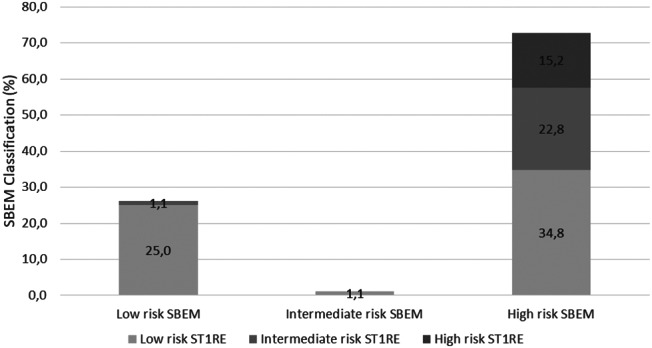
Agreement between SBEM and ST1RE classification of cardiovascular risk

Figure 1 illustrates the concordance between cardiovascular risk classifications by SBEM and ST1RE. The SBEM classification identified 26.1% of patients as Low-risk, of which 25.0% were classified as low risk by ST1RE and the remaining 1.1% as intermediate risk by ST1RE. SBEM identified 1.1% of patients as intermediate risk, which were classified as low-risk by ST1RE. The SBEM classification identified 72.8% of patients as high risk, of which 34.8% were classified as low risk by ST1RE, 22.8% as intermediate risk by ST1RE and 15.2% as high risk by ST1RE. We observed a great agreement of patients classified as “low risk” by SBEM and classified as “low risk” by ST1RE. However for patients classified as “high risk” by SBEM, only a minority was similarly classified by ST1RE, with a great proportion of patients classified as “low” or “intermediate risk” by this tool. The total agreement measured by Cohen’s Kappa coefficient was just 0.219. corresponding to a weak agreement (p ≤ 0.001).

The characteristics of high-risk populations for each tool are shown in Table 2. All 14 participants classified as “high risk” by ST1RE were also classified as “high risk” by SBEM. High risk patients by ST1RE had a significantly higher age, longer diabetes duration, higher systolic blood pressure, lower eGFR and higher prevalence of abnormal albuminuria when compared to patients classified as high risk by SBEM. The other variables in the study were did not differ the two populations with statistical significance.

**Table 2:** Characteristics of high risk participants by SBEM and ST1RE

	SBEM (n = 67)	ST1RE (n = 14)	p-value
Age (years)	38.1 (± 10.7)	50.3 (± 9.9)	0.0002*
Sex			1.000
Male	34.9 (23)	30.8 (4)	
Female	65.1 (43)	69.2 (9)	
Diabetes duration (years)	27.0 (19.0; 32.0)	34.0 (31.0; 42.0)	0.029*
Age of T1DM onset (years)	11.0 (4.0; 17.0)	16.0 (10.0; 28.0)	0.065**
Early onset T1DM (1–10 years of age)	44.8 (30)	28.6 (4)	0.375
Late onset T1DM (> 10 years of age)	55.2 (37)	71.4 (10)	
BMI (kg/m^2^)	23.4 (21.4; 28.0)	21.5 (20.4; 23.2)	0.055**
HbA1c (%)	8.6 (7.6; 10.0)	9.4 (7.8; 10.8)	0.209**
SBP (mmHg)	122.0 (110.0; 130.0)	133.0 (128.0; 144.0)	0.004**
DBP (mmHg)	80.0 (70.0; 80.0)	80.0 (78.0; 82.0)	0.226**
LDL-cholesterol (mg/dl)	90.0 (70.4; 113.4)	91.1 (71.2; 112.0)	0.925**
eGFR (CKD-EPI) (ml/min/1.73 m2	99.0 (72.0; 114.0)	69.5 (33.0; 79.0)	0.0005**
Serum creatinin (mg/dL)	0.85 (0.74; 1.03)	1.04 (0.92; 1.84)	0.0035**
Albumin-Creatinin Ratio (mg/g)			**0.004**
Normal	52.2 (35)	7.1 (1)	
Microalbuminúria	23.9 (16)	42.9 (6)	
Macroalbuminúria	23.9 (16)	50.0 (7)	
Microvascular complications (%)	83.6 (56)	100.0 (14)	0.197
Nephropathy (%)	55.2 (37)	92.9 (13)	**0.013**
Retinopathy (%)	74.6 (50)	92.9 (13)	0.175
Neuropathy (%)	13.4 (9)	28.6 (4)	0.224
Treatments			
Statins	52.2 (35)	71.4 (10)	0.244
ACE inhibitor or ARB	67.2 (45)	78.6 (11)	0.532
AAS	7.5 (5)	28.6 (4)	**0.044**
Physical activity	11.9 (8)	14.3 (2)	0.681
Smoking	4.8 (3)	7.7 (1)	0.541

Data are expressed as mean ± standard deviation or median (P25;P75) or as a percentage (%); SBP systolic blood pressure; DBP diastolic blood pressure; eGFR estimated glomerular filtration rate; BMI body mass index; T1DM type 1 diabetes mellitus; HBA1c glycated hemoglobin. *T-Student test, **Mann-Whitney test, Fisher´s Exact test were used.

**Fig. 2:**
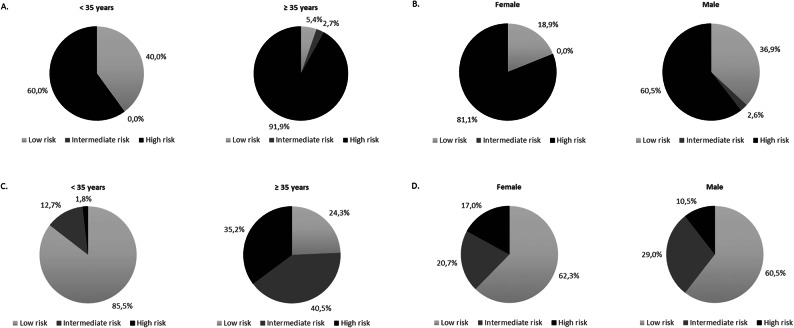
Comparison of proportions of cardiovascular risk levels according to the age and gender. (A) SBEM according to the age; (B) SBEM according to the gender; (C) ST1RE according to the age; (D) ST1RE according to the gender

The dissimilarities between these two classifications were also evident when age and gender factors were compared. While 60% of patients under 35 years were classified as high risk according to SBEM criteria (Fig. 2A), only 1.8% received this stratification risk in the ST1RE classification (Fig. 2C). This proportion increased in both estimators, reaching 91.9% of the patients aged 35 years or more in SBEM classification (Fig. 2A) and 35.8% defined by ST1RE (Fig. 2C). Considering the risk classification regarding gender, SBEM classified 81.1% of females as high risk (Fig. 2B), while 17% receive the same classification by ST1RE (Fig. 2D). Among males, the proportions are 60.5% by SBEM (Fig. 2B) and 10.5% by ST1RE, respectively (Fig. 2D).

## Discussion

Our results show a weak agreement in the 10-year cardiovascular risk estimation between SBEM and ST1RE classifications. According to SBEM criteria, 72.8% of patients were considered high-risk, while only 15.2% of patients received the same classification using ST1RE (Fig. 1) which is a considerable divergence. We identified individual factors contributing to this disagreement based on age, duration of diabetes, and gender.

Age impacted diversely on cardiovascular risk producing different interpretations about the same patients risk, even in those younger than 35 years of age. This age cutoff was suggested by the European Society of Cardiology (ESC) using Type 2 Diabetes Mellitus as a reference and produced similar distortions using the 2019 ESC risk classification [18].

Diabetes duration also brought changes to the classification. Diabetes duration over 10 years is a high-risk criterion according to SBEM recommendations [21], while for ST1RE, although included in the algorithm, disease duration alone is not sufficient to reclassify a patient from low or intermediate risk to high risk. Given the sample’s average disease duration of 23.4 (± 9.5) years, this difference seems relevant.

Gender factors also influenced the risk classification. One interesting observation in our study is that female patients were classified as high cardiovascular risk at a higher proportion than male patients, contrary to the usual expectation [24] Fig. 2). The observed discrepancy could be partially explained by the high prevalence of diabetic retinopathy in the sample, a factor that categorizes a patient as having high cardiovascular risk by SBEM but is not a variable in the ST1RE calculation as retinopathy was more prevalent among women. Women also exhibit a higher prevalence of diabetic kidney disease, as well as poorer glycemic control and slightly higher LDL levels, which could justify a higher proportion of female patients classified as high cardiovascular risk by both methods.

The divergence between the ST1RE and cardiovascular risk estimators, is not exclusive to SBEM classification since disagreements of ST1RE from other societies have been reported [17, 18]. We highlight the discordance of ST1RE with the ESC cardiovascular risk classification. In that study, a lower importance was attributed to age, and diabetes duration by ST1RE, similarly to our study [18].

In general, patients at high cardiovascular risk are recommended to use statins [22]. However, 39.1% of patients are on statin therapy, a conflicting figure when considering both classifications. Neither method predominates for patients’ cardiovascular risk analysis, and other elements are being weighed in clinical decisions regarding statin use. One possible explanation is that SBEM classification might be considered inadequate as it is more validated for type 2 diabetes, while ST1RE seems to significantly underestimate cardiovascular risk when compared to traditional stratification methods. Whether this underestimation comes closer or further from reality still remains to be seen, which suggests that cardiovascular risk stratification for type 1 diabetes patients needs to be revisited to identify more reliably those who would benefit most from interventions like statin use for the primary prevention of cardiovascular events.

The main strengths of this study were a reasonably sample size and obtention of the necessary data for clinical and laboratorial classification including a screening for diabetic chronic complications. Limitations of the study are mainly based on the cross-sectional design that impairs the extended patient follow-up for observing clinical outcomes that could estimate better the accuracy of each stratification method and the potential of selection bias given the enrollment of patients occurred in a quaternary hospital-affiliated service. This situation could explain the sample’s poor glycemic control and high rate of microvascular complications, considering that the country’s public health service attends mostly lower-income patients.

## Conclusion

The present study demonstrates low agreement between the 10-year cardiovascular event risk classification by SBEM and the classification by ST1RE for T1DM patients without established cardiovascular disease. To our knowledge, this is the first study comparing ST1RE with SBEM cardiovascular risk stratification tools. Further similar studies, with different sampling profiles, would be useful for a more comprehensive discussion of the topic.

## Data Availability

The datasets used or analyzed during the current study are available from the authors on reasonable request.

## Abbreviations

ASCVD: Atherosclerotic Cardiovascular Disease
BMI: Body mass index
CKD-EPI: Chronic kidney disease epidemiology collaboration equation
DBP: Diastolic blood pressure
eGFR: estimated glomerular filtration rate
ESC: European Society of Cardiology
HbA1c: Glycated hemoglobin
LDL: Low density lipoprotein
SBEM: Brazilian Society for Endocrinology and Metabology
SBP: Systolic blood pressure
ST1RE: Steno T1 Risk Engine
T1DM: Type 1 diabetes mellitus
T2DM: Type 2 diabetes mellitus
UFMG: Federal University of Minas Gerais
UKPDS: United Kingdom Prospective Diabetes Study

## References

[1] Livingstone SJ (2012). Risk of Cardiovascular Disease and total mortality in adults with type 1 Diabetes: Scottish registry linkage study. PLoS Med.

[2] Soedamah-Muthu SS (2006). High risk of Cardiovascular Disease in patients with type 1 Diabetes in the U.K.: a cohort study using the general practice research database. Diabetes Care.

[3] Secrest AM (2010). Cause-specific mortality trends in a large population-based cohort with long-standing childhood-onset type 1 Diabetes. Diabetes.

[4] Davis TM (1997). U.K. prospective Diabetes study 22. Effect of age at diagnosis on diabetic tissue damage during the first 6 years of NIDDM. Diabetes Care.

[5] Gerstein HC (2001). Albuminuria and risk of cardiovascular events, death, and Heart Failure in diabetic and nondiabetic individuals. JAMA.

[6] Ninomiya T (2009). Albuminuria and kidney function independently predict cardiovascular and renal outcomes in Diabetes. J Am Soc Nephrol.

[7] Goff DC (2014). 2013 ACC/AHA guideline on the assessment of cardiovascular risk: a report of the American College of Cardiology/American Heart Association Task Force on Practice guidelines. Circulation.

[8] Khaw KT, Wareham N (2006). Glycated hemoglobin as a marker of cardiovascular risk. Curr Opin Lipidol.

[9] Zambanini A, Smith MR, Feher MD. *Prediction of cardiovascular risk. Program is not suitable for diabetic patients* BMJ, 1999. 318(7195): p. 1418; author reply 1419. PMC1115793 10334766

[10] Zgibor JC (2006). Deficiencies of cardiovascular risk prediction models for type 1 Diabetes. Diabetes Care.

[11] Stevens RJ (2001). The UKPDS risk engine: a model for the risk of coronary Heart Disease in type II Diabetes (UKPDS 56). Clin Sci (Lond).

[12] Juutilainen A (2008). Similarity of the impact of type 1 and type 2 Diabetes on cardiovascular mortality in middle-aged subjects. Diabetes Care.

[13] Huxley RR (2015). Risk of all-cause mortality and vascular events in women versus men with type 1 Diabetes: a systematic review and meta-analysis. Lancet Diabetes Endocrinol.

[14] Vistisen D (2016). Prediction of First Cardiovascular Disease Event in Type 1 Diabetes Mellitus: the Steno type 1 risk engine. Circulation.

[15] Vinals C et al. *Steno type 1 risk engine and preclinical Atherosclerosis in Mediterranean individuals with type 1 Diabetes*. Diabetes Metab Res Rev, 2020: p. e3320. 10.1002/dmrr.3320 32239693

[16] Llaurado G (2019). Arterial stiffness is highly correlated with the scores obtained from the Steno type 1 risk engine in subjects with T1DM. PLoS ONE.

[17] Boscari F (2020). Performance of the Steno type 1 risk engine for Cardiovascular Disease prediction in Italian patients with type 1 Diabetes. Nutr Metab Cardiovasc Dis.

[18] Tecce N (2020). Evaluation of cardiovascular risk in adults with type 1 Diabetes: poor concordance between the 2019 ESC risk classification and 10-year cardiovascular risk prediction according to the Steno type 1 risk engine. Cardiovasc Diabetol.

[19] Cano A et al. *Utility of insulin resistance in estimating Cardiovascular risk in subjects with type 1 Diabetes according to the scores of the Steno type 1 risk engine*. J Clin Med, 2020. 9(7). 10.3390/jcm9072192 PMC7409001 32664522

[20] Jensen MT (2019). Echocardiography improves prediction of major adverse cardiovascular events in a population with type 1 Diabetes and without known Heart Disease: the Thousand & 1 study. Diabetologia.

[21] Bertoluci MC (2017). Brazilian guidelines on prevention of Cardiovascular Disease in patients with Diabetes: a position statement from the Brazilian Diabetes Society (SBD), the Brazilian Cardiology Society (SBC) and the Brazilian endocrinology and Metabolism Society (SBEM). Diabetol Metab Syndr.

[22] Izar M, Faludi FF, Araújo A, Valente D, Bertoluci F, Manejo M (2021). Do risco cardiovascular: dislipidemia. Diretriz Oficial Da Sociedade Brasileira De Diabetes (2022).

[23] Landis JR, Koch GG (1977). The measurement of observer agreement for categorical data. Biometrics.

[24] Booth GL, Kapral MK, Fung K, Tu JV (2006). Relation between age and Cardiovascular Disease in men and women with Diabetes compared with nondiabetic people: a population-based retrospective cohort study. Lancet.

